# Multiplexed Visualization Method to Explore Complete Targeting Regulatory Relationships Among Circadian Genes for Insomnia Treatment

**DOI:** 10.3389/fnins.2022.877802

**Published:** 2022-07-01

**Authors:** Tao Li, Zhenyu Liu, Yitong Wang, Dongshi Zuo, Shenyuan Wang, Haitao Ju, Shichao Wang, Yanping Xing, Yu Ling, Chunxia Liu, Yanru Zhang, Huanmin Zhou, Jun Yin, Junwei Cao, Jing Gao

**Affiliations:** ^1^Inner Mongolia Key Laboratory of Bio-manufacture, College of Life Sciences, Inner Mongolia Agricultural University, Hohhot, China; ^2^Inner Mongolia Autonomous Region Key Laboratory of Big Data Research and Application of Agriculture and Animal Husbandry, College of Computer and Information Engineering, Inner Mongolia Agricultural University, Hohhot, China; ^3^Department of Neurosurgery, Beijing Hospital, Beijing, China; ^4^Department of Neurosurgery, Affiliated Hospital of Inner Mongolia Medical University, Hohhot, China; ^5^Clinical Genetic Laboratory, First Hospital of Hohhot, Hohhot, China

**Keywords:** melatonin synthesis, circadian genes, insomnia, transfer information, dynamic sequential omics, visualization method

## Abstract

Understanding the complete map of melatonin synthesis, the information transfer network among circadian genes in pineal gland, promises to resolve outstanding issues in endocrine systems and improve the clinical diagnosis and treatment level of insomnia, immune disease and hysterical depression. Currently, some landmark studies have revealed some genes that regulate circadian rhythm associated with melatonin synthesis. However, these studies don't give a complete map of melatonin synthesis, as transfer information among circadian genes in pineal gland is lost. New biotechnology, integrates dynamic sequential omics and multiplexed imaging method, has been used to visualize the complete process of melatonin synthesis. It is found that there are two extremely significant information transfer processes involved in melatonin synthesis. In the first stage, as the light intensity decreased, melatonin synthesis mechanism has started, which is embodied in circadian genes, *Rel, Polr2A, Mafk*, and *Srbf1* become active. In the second stage, circadian genes *Hif1a, Bach1, Clock, E2f6*, and *Per2* are regulated simultaneously by four genes, *Rel, Polr2A, Mafk*, and *Srbf1* and contribute genetic information to *Aanat*. The expeditious growth in this technique offer reference for an overall understanding of gene-to-gene regulatory relationship among circadian genes in pineal gland. In the study, dynamic sequential omics and the analysis process well provide the current state and future perspectives to better diagnose and cure diseases associated with melatonin synthesis disorder.

## 1. Introduction

In 2017, three scientists who made outstanding contributions to the research of circadian rhythm shared the Nobel Prize in physiology or medicine. The three scientists were Jeffrey C. Hall, Michael Rosbash, and Michael W. Young. After years of research, they discovered the molecular mechanism controlling circadian rhythm. The rhythm gene Doubletime (high expression at night and low expression during the day) encodes DBT protein and activates the Period gene to express PER protein. PER protein enters the nucleus under the transport of TIM protein encoded by Timeless gene and activates the rate limiting enzyme gene *Aanat* (Kloss et al., 1998). The research result has great applications in the field of medical and health, such as the treatment of insomnia. People with insomnia may have disorder in the synthesis and function of three proteins, and this research result will become a guide for finding targeted drugs and point out the direction for future research. Among the more than 100 human mental disorders reported in WHO website (https://www.who.int/teams/mental-health-and-substance-use), sleep disorder has become the second most common mental disorder around the world. One out of every three people has sleep problems, and sleep disorders tend to be younger and more common. Therefore, it is very urgent to find more molecular mechanisms that control the circadian rhythm and find targeted drugs to treat sleep disorders.

Current research has found that sleep and wakefulness are related to the cyclic rhythm of the pineal gland, and the cyclic rhythm of the pineal body is regulated by related rhythm genes. So far, researchers have found that the secretion of melatonin is closely related to sleep, and proved that some genes play an important role in insomnia. For example, in patients with abnormal sleep caused by inflammation, Morris et al. found that the expression of the key circadian rhythm regulator *Clock* gene was abnormal (Morris et al., 2018). In patients with traumatic brain injury with sleep disorders, Niu et al. found that *Clock, Per2*, and *Bmal1* rhythm genes were abnormally expressed (Niu et al., 2019). And Mcclung found that the antidepressant and insomnia drug fluoxetine can change the expression levels of rhythm genes *Clock, Bmal1*, and *Npas2* (Mcclung, 2007). Dijk and Archer discovered that the rhythm gene *Per3* plays an important role in sleep and wakefulness (Dijk and Archer, 2010). In addition, the expression pattern of a circadian rhythm gene named *Per2* that is affected by light was studied. It was found that the *Per2* gene oscillates approximately 1/4 times per day in the circadian rhythm, while the non-imaging visual system is involved in the human circadian rhythm (Cajochen et al., 2006; van der Veen et al., 2020). Dutta et al. found that the expression levels of serotonergic genes were up-regulated in rice bran and sarcodon aspratus. Rice bran and sarcodon aspratus supplements can be used as food materials to promote sleep (Dutta et al., 2021). Dmitrzak-Weglarz et al. identified dysfunction of molecular regulation of the melatonin biosynthetic pathway on *Aanat*, rs8150 and rs3760138 and detected serotonin, *Aanat, Aamt* and melatonin in serum. The results of this experimental analysis revealed the only pathways and genes that were altered after agomelatine treatment in clinical models and melatonin treatment in cell culture models. And the immunomodulatory effects of melatonin preparations on depression were demonstrated in both models (Dmitrzak-Weglarz et al., 2021). Gobbi and Comai found that activation of the *MT1* receptor is primarily participated in the regulation of rapid eye movement (REM) sleep. However, the *MT2* receptor can selectively increases non-REM (NREM) sleep. Experiments in mutant mice demonstrated that when the *MT1* gene was knocked out, NREM sleep was increased and REM sleep was decreased. However, NREM sleep was reduced in mice knocked out of the *MT2* gene. Therefore, *MT1* and *MT2* have selective ligand properties of agonists and antagonists, and they play an important role in sleep regulation, which may help to study the potential of sleep treatment (Gobbi and Comai, 2019). Due to increasing age, SCN, biological clock, circadian rhythm, etc. will be affected. Jagota and Kalyani experimentally demonstrated that the regularity and magnitude of cyclic changes in SCN serotonin due to increasing age were restored, and thus found that the correlation between melatonin and age was lost (Jagota and Kalyani, 2010). Kirsz et al. found that *Oxa* gene can promote the expression of *Aanat* gene (Kirsz et al., 2020). Day length and hypothalamic Oxa non-light signals can modulate the endocrine activity of ovine PG. Both the *Oxa* gene and melatonin are involved in the regulation of the sleep-wake system. Since *Cry2* circadian genes are involved in the regulation of nocturnal oscillators. Further studies by Lavebratt et al. found that the *Cry2* locus is associated with susceptibility to depression, and its mechanism of action involves dysregulation of *Cry2* expression (Lavebratt et al., 2010). Nishiyama and Hirai found that agonist washout and forskolin stimulation resulted in enhanced oscillatory expression of *Rev, Erba*, and *Bmal1* genes. The results of these experiments provide new ideas for the study of the duration-dependent effects of Lamerton on the expression of *Clock* genes in INS-1 cells, and contribute to a better understanding of its role *in vivo* (Nishiyama and Hirai, 2014). Hanish and Han found that it hard for teenagers to fall asleep with PAX6+/-. The finding seems to be a useful measure used for treatment of insomnia patients with PAX6-insufficient and/or melatonin of low concentration (Hanish and Han, 2018). The experiments by Larion et al. showed that the mRNA expression level of clock-controlled gene was increased significantly over baseline at 5 am and 11 pm in all subjects. However, the mRNA expression level of *Alas1, Alas2*, and *Pbgd* were increased only at 11 pm in subjects with active acute intermittent porphyria. These results may provide evidence for disturbances of circadian markers in subjects with active *Aip*. The *Clock* genes relating to circadian markers were of prognostic value in patients with intermittent porphyria (Larion et al., 2013). Schulz and Steimer proved that the biological clocks are influenced by not only non-pharmacological (light therapy, sleep deprivation, rhythm therapy) but also pharmacological (lithium, antidepressants, agomelatine). The results may indicate circadian system play an important role in sleep disorders (Schulz and Steimer, 2009). Sha et al. showed that there was an association between the improvement of circadian rhythm related behavior in HIBD animals and mir-325 and LHX3. Their findings also suggested that the mir-325-lhx3 axis is not only linked to regulate circadian rhythms but response to identify the potential therapeutic targets in neonatal HIBD patients with circadian rhythm disorders (Sha et al., 2021). The experiments by Williams et al. show that haploid deficiency of Rai1 in SMS fibroblasts and mouse hypothalamus leads to transcriptional imbalance of circadian clock and changes in the expression and regulation of circadian genes, including *Per2, Per3, Cry1, Bma1*, etc. Besides, the experimental data indicated that an abnormal sleep-wake cycle occurs when a disrupted circadian rhythm which is caused by heterozygous mutation of *Rai1*. Moreover, the experimental data also shown that an abnormal sleep-wake cycle can lead to dependent cognitive performance and abnormal eating patterns. Therefore, we has evolved that *Rai1*, as a positive transcriptional regulator of *Clock*, play important role for regulation of sleep-wake cycle by circadian oscillators (Williams et al., 2012).

Currently, the landmark studies might partly reveal the molecular mechanism in controlling circadian rhythm. It was visible in fixed regulation process or information transfer process. However, there is no relevant research on providing the time evolution map of regulation information among circadian genes. In this paper, authors first give the time evolution map of regulation information among circadian genes. It shows the molecular mechanism in information transfer among circadian genes completely, comprehensively and objectively. In this way, It's obvious to confirm that the targeting regulatory relations among circadian genes. It provides a theoretical guarantee for finding targeted drugs.

According to the 24-h circadian rhythm, we designed new omics scheme-dynamic sequential RNA sequencing. Using this experimental scheme, 480 dynamic time series expression profiles covering the whole rhythm cycle were obtained. The scheme resolves the pseudo time problem in single-cell RNA sequencing (Nawy, 2014; Ji and Ji, 2016). In order to process and analyze the dynamic time series expression profiles, a new image method is introduced based on transfer information theory (Schreiber, 2000), markov model (Kalpazidou, 2006), network decomposition theory and dynamic network comparison model (Eun and Shroff, 2005). By using the method, we can explore complete targeting regulatory relations among rhythmic genes, especially the changing trend of the regulator relationship with time in melatonin synthesis process.

## 2. Materials and Methods

### 2.1. Technology Strategy

**Figure 1:**
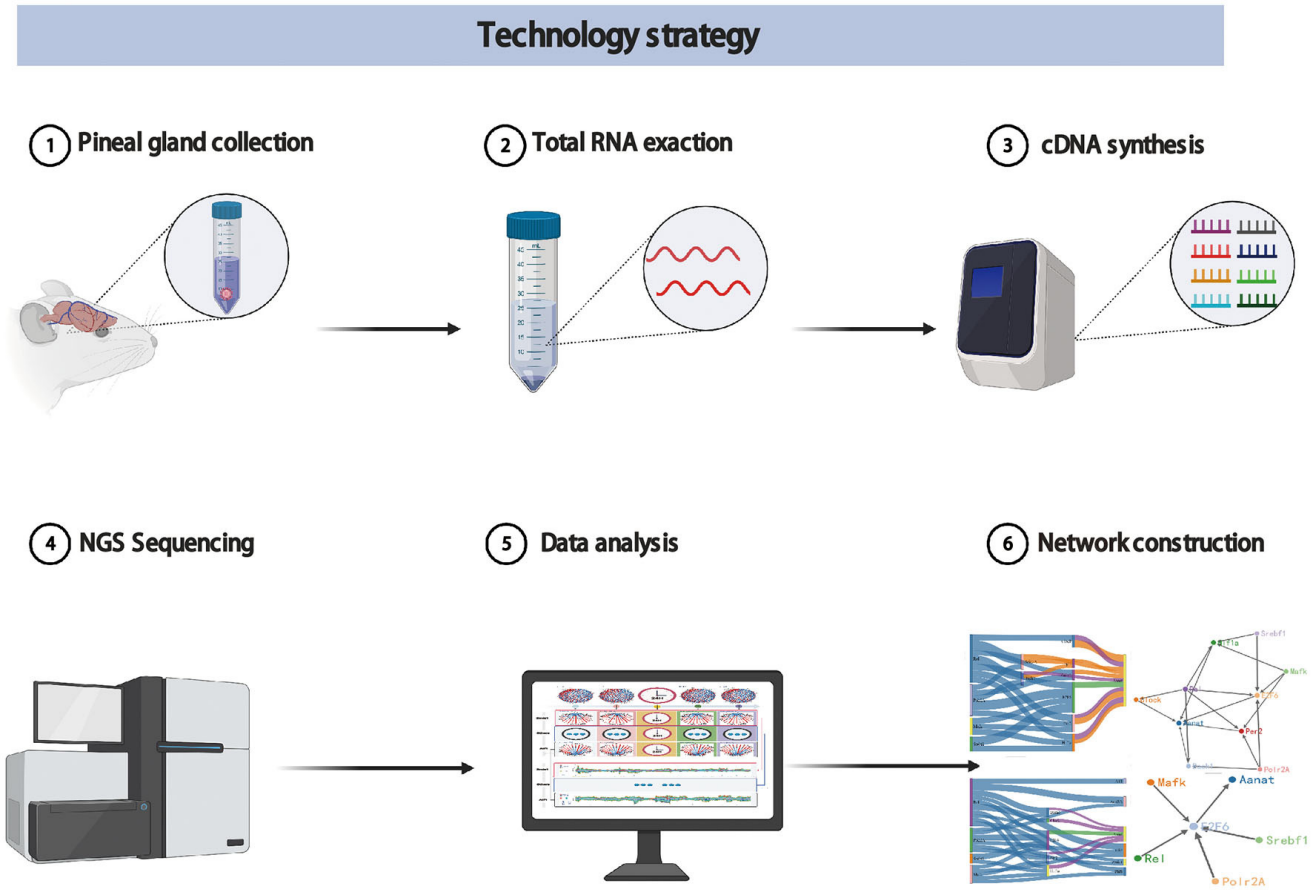
The technology strategy for experimental protocols, data analysis methods, and visualization strategies.

During a day and night cycle, the synthesis and secretion of melatonin in pineal gland shows a periodic pattern. This pattern is a very complex process of information transfer. The information transfer with certain regulations may determine melatonin synthesis and secretion in pineal gland (Binkley, 1983). In order to provide a complete map of melatonin synthesis, we designed a new spatial view, time-network relations-variables, to explore the trend of network relationship with time, that is, the regulation information between any two genes in the network how to change with time. In addition, we also design new dynamic time-series experimental protocols, data analysis methods and visualization strategies. The technology strategy is shown in Figure 1.

### 2.2. Rat Pineal Gland Collection, Total RNA Extraction, and cDNA Library Construction

A total of 480 male rats, aged 8 weeks, with an average body-mass index of 180 g, were selected from the rat aquatic breeding farm in Qingdao, Shandong Province. All experimental rats were kept in a 100 square meters independent rat room for 2 weeks (free feeding, free drinking, and free lighting). Each experimental rat is kept in a independent small cage. This strategy ensures that rats are not disturbed each other in the experiments. In complete circadian rhythm cycle, the rat pineal gland was sampled every 3 min from 7:00 a.m. on November 15, 2020 to 7:00 a.m. on November 16, 2020. It was carried out continuously for 24 h until the end of the experiment. 21 postgraduate students, participate in rat pineal gland collection, are divided into three groups. Each group independently perform according to the scheduled time with the following 7 steps. (1) Take rat from the rat room to the test bench (2) Euthanize rat (3) Open the skull, and take out the brain tissues (4) Isolate the rhythm center-pineal gland (5) Second microstructure identification (6) Remove rat pineal gland and put it in a 2 ml Corning Freezer Tube (7) Label the sample with detailed information and put in liquid nitrogen immediately. Before the formal experiment, we carried out a month-long pre-experiment. This rule ensures that the harvesting of the pineal glands is more accurate and reliable. After pineal gland collection, the total RNA was extracted using the Biomend RNApure Rapid RNA Kit (RA103-02). The total RNA extraction results were detected by Agilent Bioanalyzer 2100. According to the manufacturer's instructions in NEBNext^®^ UltraTM RNA Library Prep Kit (NEB, USA), the 480 sequencing libraries were generated in our lab. Meanwhile, we added index codes to each library sample. Each Library's quality was also assessed on the Agilent Bioanalyzer 2100 system.

### 2.3. Dynamic Time-Series Expression Profiles Obtained by RNA Sequencing

RNA sequencing means to obtain the total mRNA from specific tissues or cells in a specific state. It can find differentially expressed genes on insecticide-treated and control samples. The differential expression genes can revealed the molecular elements of biological phenomena. For example, it's already used liberally to the study of identify the new biomarkers, reveal the mechanisms of inhibition of growth of carcinoma, and shed light on metastasis and drug resistance (Lappalainen et al., 2013; Bai et al., 2018; Demircioglu et al., 2019; Mohammadi et al., 2019; Mahadevaiah et al., 2021). In this work, RNA sequencing can be used for the detection of gene mRNA expression level in rat pineal gland. The index-coded samples constructed in 2.2 was clustered on a cBot Cluster Generation System based on the manufacturer's recommendations in TruSeq PE Cluster Kit v3-cBot-HS. After cluster generation, the libraries were sequenced on the BGI DNBSEQ-T7RS platform and 125 bp/150 bp paired-end reads were generated. Therefore, 480 dynamic time-series expression profiles were obtained in complete circadian rhythm cycle. The information such as sequencing platform, bulk RNA-seq, number of reads per sample, and quality metrics of RNA sequencing datasets has been uploaded to Sequence Read Archive (SRA) (https://www.ncbi.nlm.nih.gov/sra) (SRR18934928 ~ SRR18935407).

### 2.4. Transfer Information Theory

Entropy in general represents the uncertainty, ambiguity, and disorder of a stochastic process. Based on the axioms imposed on probability distributions whose entropic quantities we want to measure, we can identify various useful entropic functions: Shannon entropy, Renyi entropy, Tsallis entropy, conditional entropy, permutation entropy, approximate entropy, and transfer entropy (Gray, 1990; Edwards, 2008). Transfer entropy (*TE*) is a model-free method that is able to detect causality in the form of transfer information between two random processes. Besides this, It also can recognize direct or indirect causality without assuming any basic model (Schreiber, 2000; Vicente et al., 2011). Given the past time evolution of process *Y*, *TE* from system *X* to *Y* is the amount of Shannon uncertainty reduction in the future time evolution of *Y* including the knowledge of the past evolution of *X*. *TE* formula (from *X* to *Y*) can be written as follows:


(1) $$\begin{array}{r} TE_{X\to Y}=\underset{1\leq n\leq m}{\sum }p\left(y_{n+1},y_{n}^{\left(l\right)},x_{n}^{\left(k\right)}\right)\log\frac{p\left(y_{n+1}|y_{n}^{\left(l\right)},x_{n}^{\left(k\right)}\right)}{p\left(y_{n+1}|y_{n}^{\left(l\right)}\right)} \end{array}$$

where *X* = (*x*_1_, *x*_2_, …, *x*_*n*_)and*Y* = (*y*_1_, *y*_2_, …, *y*_*n*_), 1 ≤ *n* ≤ *m, n, m* ∈ *N* are time evolutions of process, respectively. $x_{n}^{\left(k\right)}=\left(x_{n},\ldots ,x_{n-k+1}\right)\text{ and }y_{n}^{\left(l\right)}=\left(y_{n},\ldots ,y_{n-l+1}\right)$ are *k*-order and *l*-order processes *X* and *Y*, *k* and *l* are the adjustable parameters. In the *TE* calculations process, adjustable parameters *k* and *l* set to 1 mean that the time evolution process *X* and *Y* are Markov process. The Markov process: Suppose {*X*(*t*), *t* ∈ *T*} as a stochastic process, *E* as its state space, for any *t*_1_ ≤ *t*_2_ ≤, …, *t*_*n*_, ≤, *t*, *x*_1_, *x*_2_, …, *x*_*n*_, *x* ∈ *E* the conditional distribution function of a random variable *X*(*t*) under known variable *X*(*t*_1_) = *x*_1_, …, *X*(*t*_*n*_) = *x*_*n*_ is only related to *X*(*t*_*n*_) = *x*_*n*_ rather than *X*(*t*_1_) = *x*_1_, …, *X*(*t*_*n*−1_) = *x*_*n*−1_. Therefore, in our analysis the *k* and *l* parameters are set to 1 (Kalpazidou, 2006).

Similarly, the calculation formula for the transfer entropy from *Y* to *X* is shown in formula 2,


(2) $$\begin{array}{r} TE_{Y\to X}=\underset{1\leq n\leq m}{\sum }p\left(x_{n+1},x_{n}^{\left(k\right)},y_{n}^{\left(l\right)}\right)\log\frac{p\left(x_{n+1}|x_{n}^{\left(k\right)},y_{n}^{\left(l\right)}\right)}{p\left(x_{n+1}|x_{n}^{\left(k\right)}\right)} \end{array}$$

where the parameters in formula 2 is exactly the same to that in formula 1.

In theory, due to the past states of process *X*, transfer entropy described the reduction uncertainty in process *Y*. *TE*_*X*→*Y*_−*TE*_*Y*→*X*_ indicates the degree of influence for *X* on *Y*. Therefore, if it is greater than zero, we can determine *X* affects *Y*, else *Y* affects *X*. From this, the transfer entropy difference (*TD*) is defined by,


(3) $$\begin{array}{r} TD\left(X,Y\right)=TE_{X\to Y}-TE_{Y\to X} \end{array}$$

where *TE*_*X*→*Y*_ and *TE*_*Y*→*X*_ are calculated by formula 1 and formula 2.

In brief, *TD*(*X, Y*) provides a powerful information theoretic measurement of directed information flow between time series variables. The asymmetric information brings about the causal driving relationship. The transfer information theory has been widely used in various fields (Castro et al., 2019; Yao and Li, 2020; Kim et al., 2021; Parente and Colosimo, 2021). In this work, we infer the quantitative description of regulation information between any two rhythmic genes by *TE* and *TD*. Furthermore, we explore the time evolution map of regulation information among circadian genes based on the regulatory relations. Ultimately, we provide a complete visualization of melatonin synthesis, especially the key targeting regulation relationship among circadian genes.

## 3. Results and Discussions

### 3.1. Determination of Circadian Genes Associate With Melatonin Synthesis

**Figure 2:**
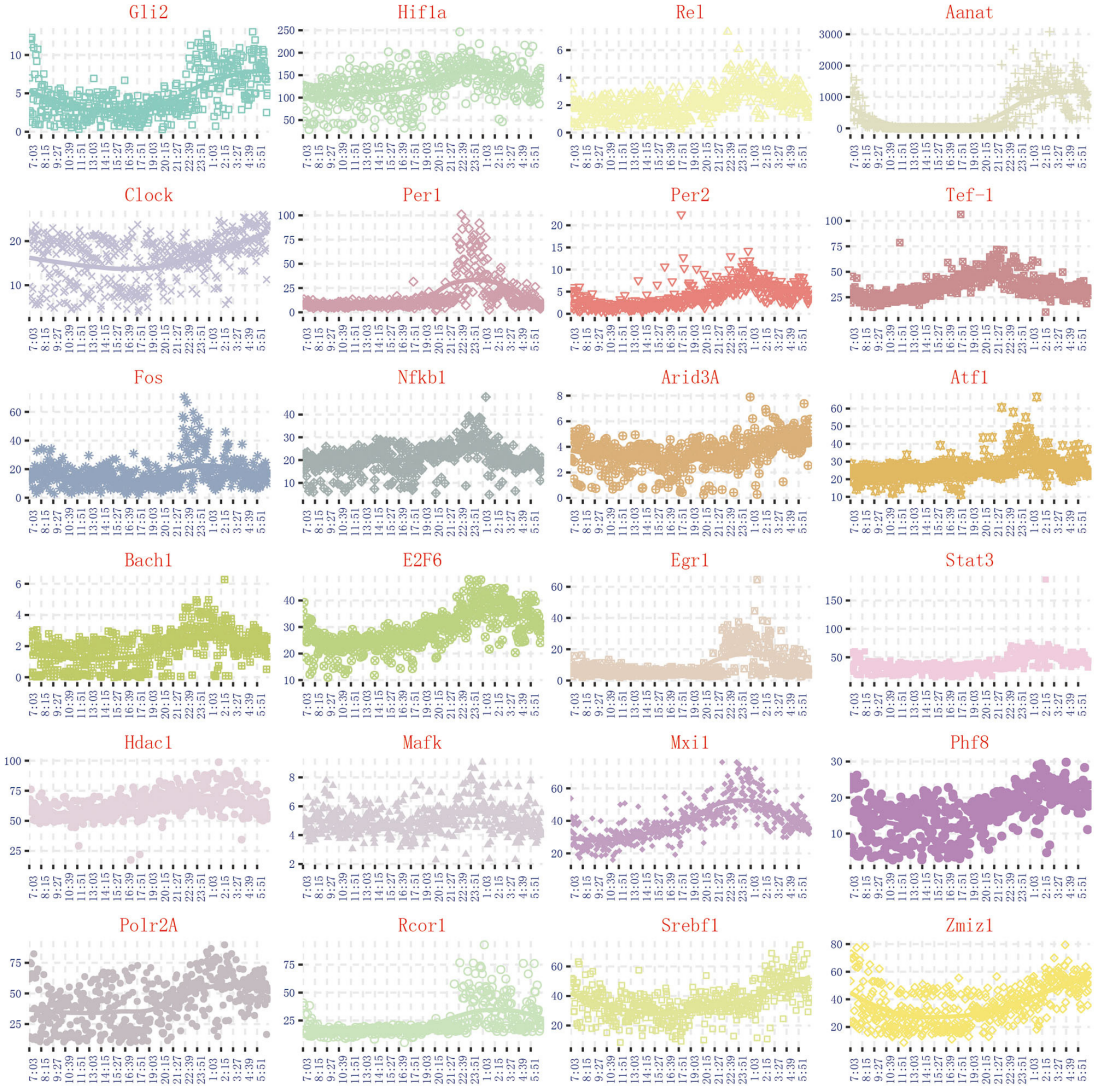
The expression trend of 24 genes related to pineal circadian rhythm regulation.

Through the literature excavation, the topic took pineal circadian rhythm regulation as a breakthrough point, revealed that 122 genes are correlated with melatonin secretion (Rouillard et al., 2016). The synthesis and secretion of melatonin have obvious circadian rhythm, which is inhibited in the day and activated at the night. Due to the synthesis and secretion of melatonin are highly correlated with N-acetyltransferase (*Aanat*) activity, we can select circadian genes based on the same expression trend of *Aanat* in the 480 dynamic time-series expression profiles. By comparing the expression trend of *Aanat* with others 121 genes in 480 dynamic time-series expression profiles, it was finally determined 24 circadian genes are highly consistent with melatonin rhythm cycle. The expression trend of 24 circadian genes is shown in Figure 2. The 24 genes are *Aanat, Gli2, Hif1a, Rel, Clock, Per1, Per2, Tef-1, Fos, Nfkb1, Arid3A, Atf1, Bach1, E2F6, Egr1, Stat3, Hdac1, Mafk, Mxi1, Phf8, Polr2A, Rcor1, Srebf1, Zmiz1*. Some important genes, such as *Isl1* and *Creb*, are considered to be important regulatory genes of *Aanat* (Simonneaux et al., 2006; Qiu et al., 2019). However, these genes are completely not expressed in 480 dynamic expression profile experimental data (fpkm <1), so they are not discussed in our work.

### 3.2. Determination of Targeting Relationship Among 24 Circadian Genes

**Figure 3:**
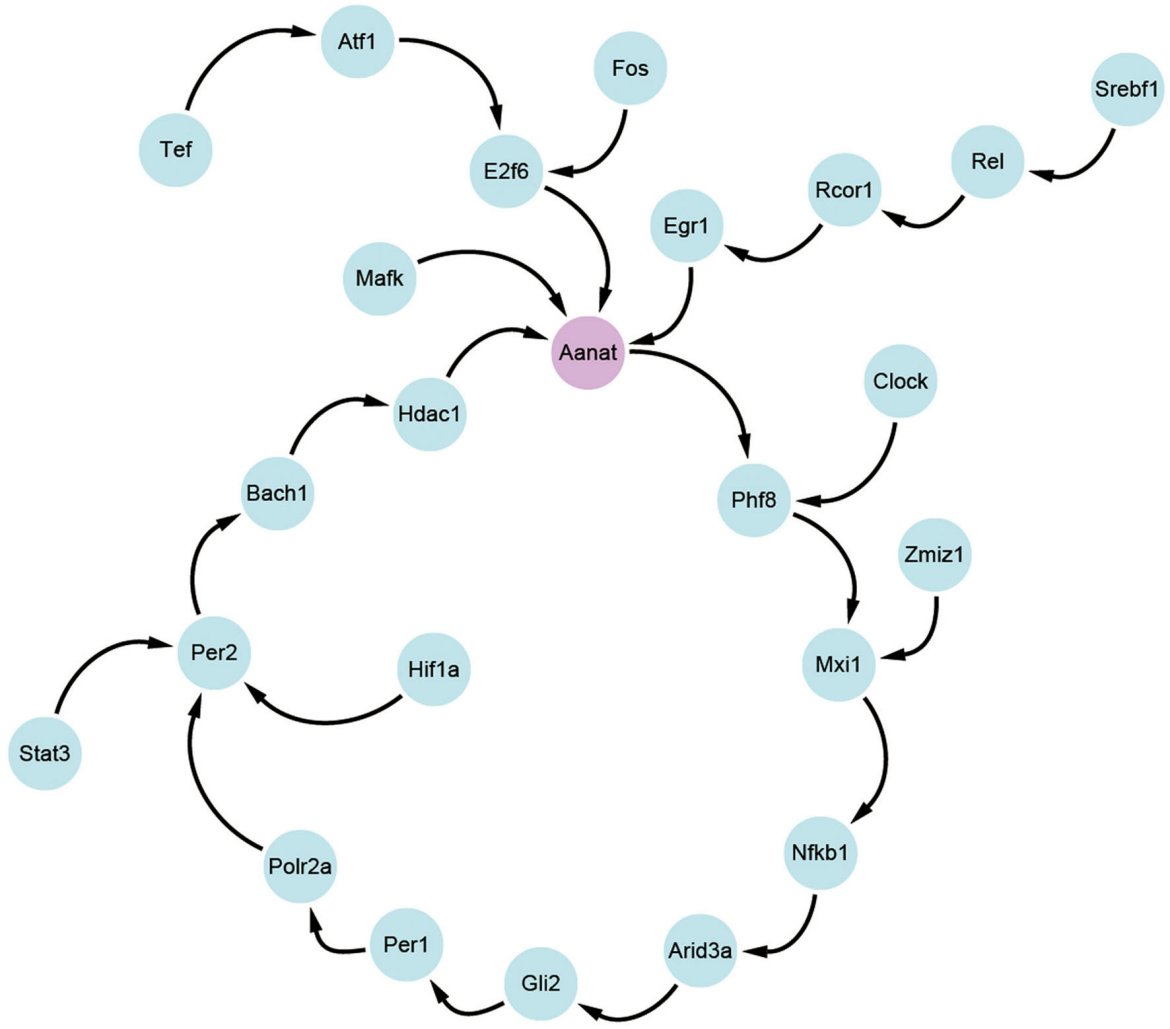
The targeting relationship between 24 circadian genes.

Based on the transfer information theory, the transfer entropy among 24 circadian genes is calculated, and the maximum value between each two genes is retained. By using cytoscape software (Shannon et al., 2003), transfer information among the 24 genes is visualized in Figure 3. From the figure, it is clear that N-acetyltransferase gene (*Aanat*) is a potential target gene for melatonin rhythm regulation in rat pineal gland. The conclusion is consistent with the results in published studies (Binkley, 1983; Chansard et al., 2005; Fukuhara et al., 2005; Simonneaux et al., 2006; Zilberman-Peled et al., 2007).

### 3.3. The Complete Information Transfer Network Among 24 Circadian Genes

**Figure 4:**
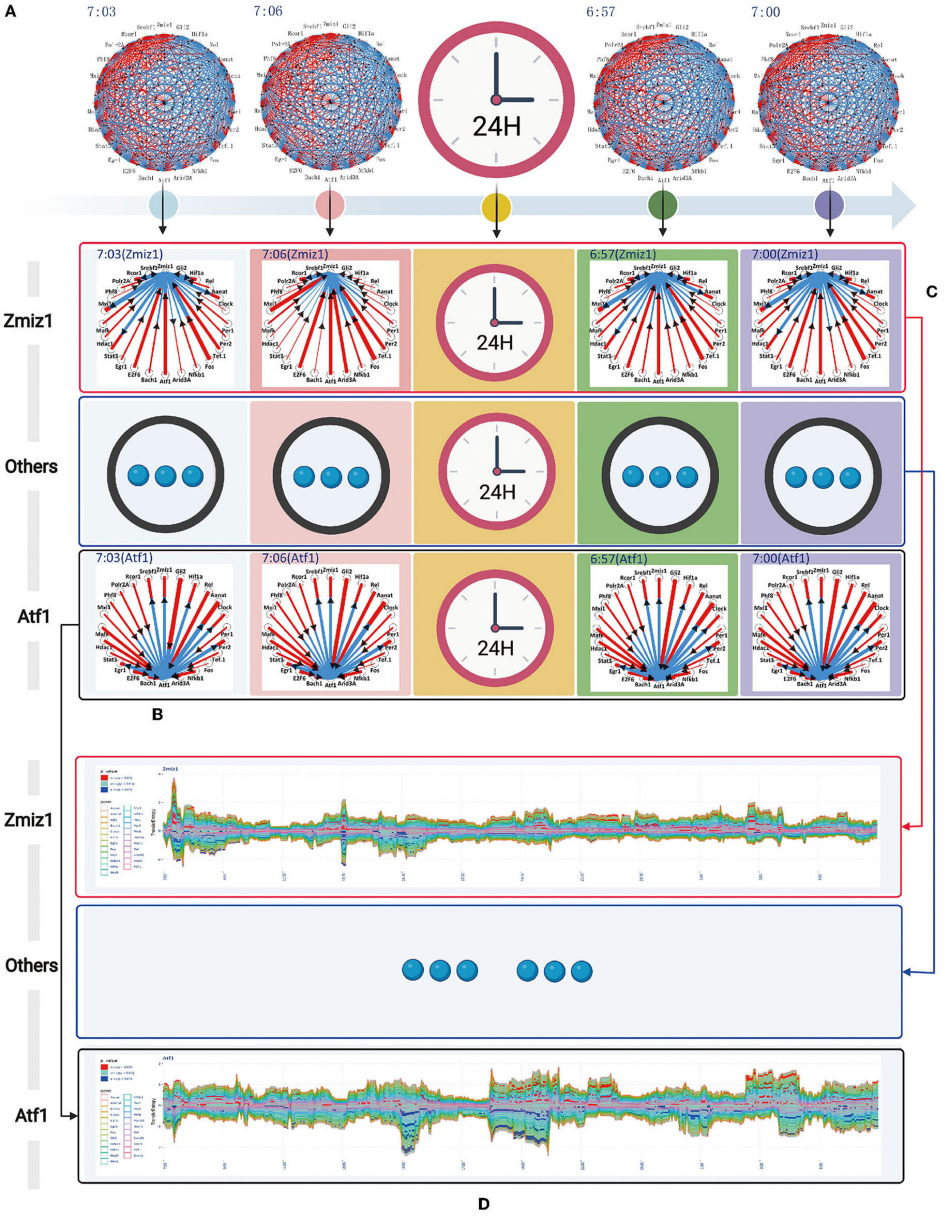
The complete information transfer network among 24 rhythmic genes **(A)** The transfer information network among 24 rhythm genes for 480 dynamic timing samples **(B)** The column represents the information transfer network among 24 rhythm genes (7:03) can be split into a series of information transfer networks for each rhythm gene to the other 23 rhythm genes **(C)** The horizontal represents the time-dependent relationship for rhythm gene (Zmiz1) to the other 23 rhythm genes. **(D)** A flow graph represents the time-dependent variation of one-to-many relationship (gene Atf1 to the other 23 circadian genes). The red color blocks, indicating that the central gene significantly regulate other genes (*P* value < 0.05). The blue color blocks, indicating that the central gene is significantly regulated by other genes (*P* value < 0.05).

The transfer information between any two genes is encoded by vector coding, for example, the transfer information is described by the connecting line segment between two genes (*X* and *Y*). The length of connecting line segment is represented by two colors, red and blue. The length of red and blue are the size of the transfer entropy *TE*_*X*→*Y*_ and *TE*_*Y*→*X*_ respectively. The width of connecting line segment is represented by *TE*_*X*→*Y*_ + *TE*_*Y*→*X*_. The direction of the connecting line segment is represented by an arrow. We used *TD*(*X, Y*) = *TE*_*X*→*Y*_ − *TE*_*Y*→*X*_ to define the direction of the connecting line segment. If *TD*(*X, Y*)> 0, indicates the direction of information transfer is from red to blue. Conversely, *TD*(*X, Y*) < 0 indicates the direction of information transfer is from blue to red. According to the newly designed vector encoding method, we obtained the transfer information network among 24 circadian genes in one time sample, as represented in Figure 4A. All the transfer information networks for 480 dynamic timing samples are shown in the Appendix 1.

The information transfer network among 24 circadian genes in each temporal state of the network is a many-to-many relational network topology. According to the basis of network decomposition theory, the many-to-many relationships can all be split into a series of one-to-many relationships. Therefore, for 7:03 time state, we split the information transfer network among 24 circadian genes into a series of information transfer network for each rhythm gene to the other 23 circadian genes. The column represents the splitting processes for each time state in throughout 24-h circadian cycle (see Figure 4B). All the splitting results for 480 dynamic timing samples are shown in the Appendix 2. The horizontal represents the time-dependent relationship for gene *Zmiz1* to the other 23 circadian genes (see Figure 4C). All the time-dependent relationships for 24 circadian genes are shown in the Appendix 3.

**Figure 5:**
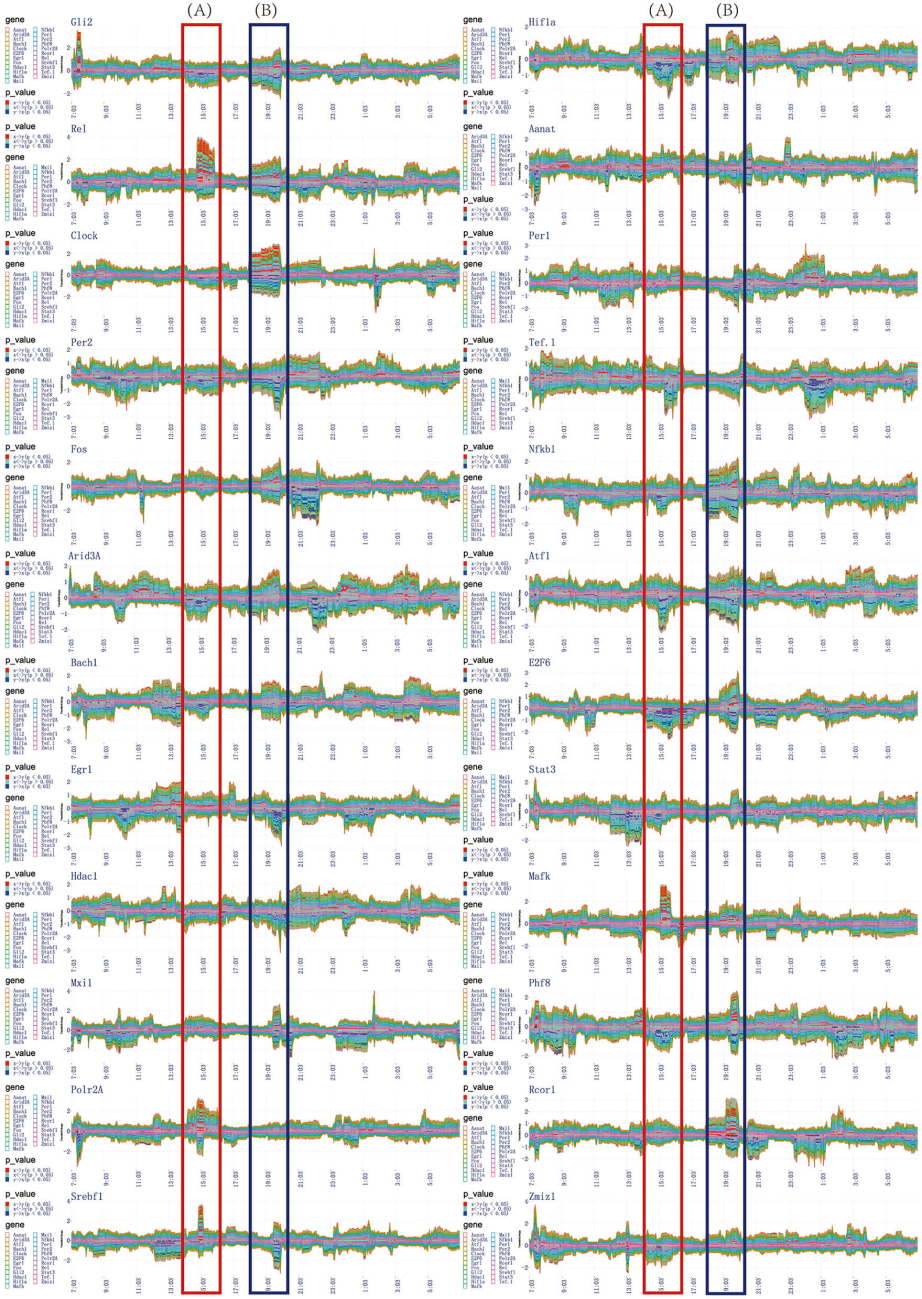
The time-dependent variation of transfer information among 24 circadian genes in 24 h. **(A)** The first extremely significant information transfer process occurs in 14:00–16:00. **(B)** The second extremely significant information transfer process occurs in 18:00–20:00.

In order to represent the time-dependent variation of one-to-many relationship, we adopted a modified flow graph design to improve the visualization scheme for one-to-many relationship. For example, given a transfer network with 3 node variables *A*, *B*, and *C*, we choose node *B* as central variable, the transfer entropy *TE*_*B*→*A*_, *TE*_*B*→*C*_ are stacked above the time axis in order, and the transfer entropy *TE*_*A*→*B*_, *TE*_*C*→*B*_ are stacked below the time axis in order. Based on this coding model, we obtained the time-dependent relationship for gene Zmiz1 to the other 23 circadian genes, as represented in Figure 4D. All the time-dependent relationship for 24 circadian genes are shown in the Figure 5. In addition, we color-coded the transfer information with significant changes (*P* < 0.05). Above the time axis, we marked with red color blocks, indicating that the central gene significantly regulate other genes. Below the time axis, we mark with blue color blocks, indicating that the central gene are significantly regulated by other genes (refer to Figure 4D).

In the flow graph of one-to-many relationship, we can clearly get the time periods where the transfer information with significant changes (*P* < 0.05). Same time periods marked by red blocks illustrate the central gene significantly regulate other genes. Others marked by blue blocks illustrate the central gene are significantly regulated by other genes. In order to be more clearly and intuitively represent the regulatory relationship, information transfer relationship, and target relationship among 24 circadian genes, three different coding methods are used to integrate the transfer information with significant changes.

**Figure 6:**
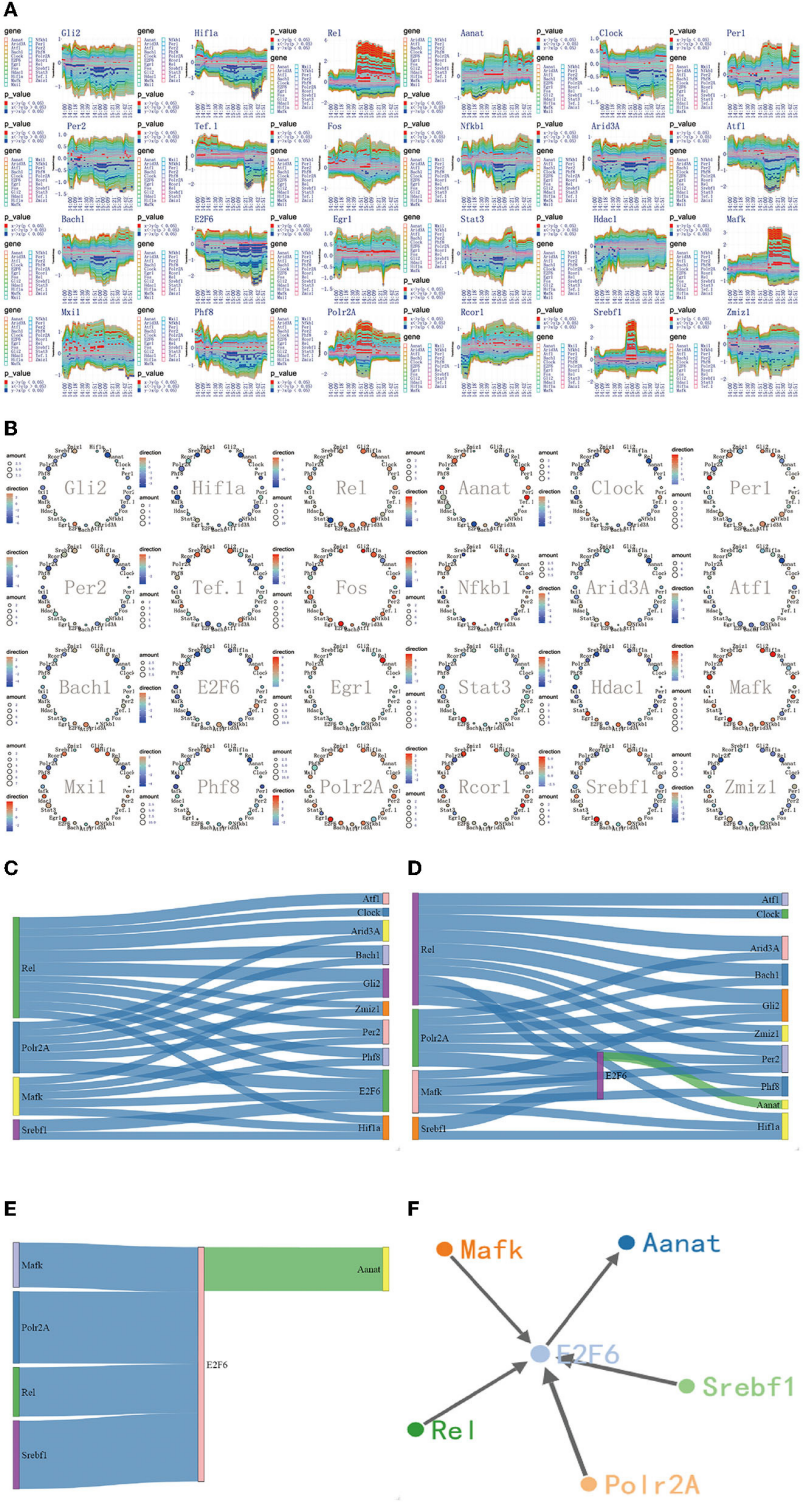
The regulatory mechanism of first extremely significant information transfer process in 14:00-16:00. **(A)** The time-dependent variation of transfer information among 24 circadian genes in 14:00–16:00. **(B)** The complete regulatory relationship among 24 circadian genes in 14:00–16:00. **(C)** The target genes regulated by four 4 promoter genes, *Rel, Polr2A, Mafk*, and *Srbf1*. **(D)** The rate-limiting gene *Aanat* is activated by *E2f6* in 14:00–16:00. **(E)** The main mechanism for information transfer process in 14:00–16:00, *E2f6* is regulated simultaneously by 4 promoter genes, and contributes genetic information to *Aanat*. **(F)** The targeting regulatory relations between promoter genes and *Aanat* in 14:00–16:00.

**Figure 7:**
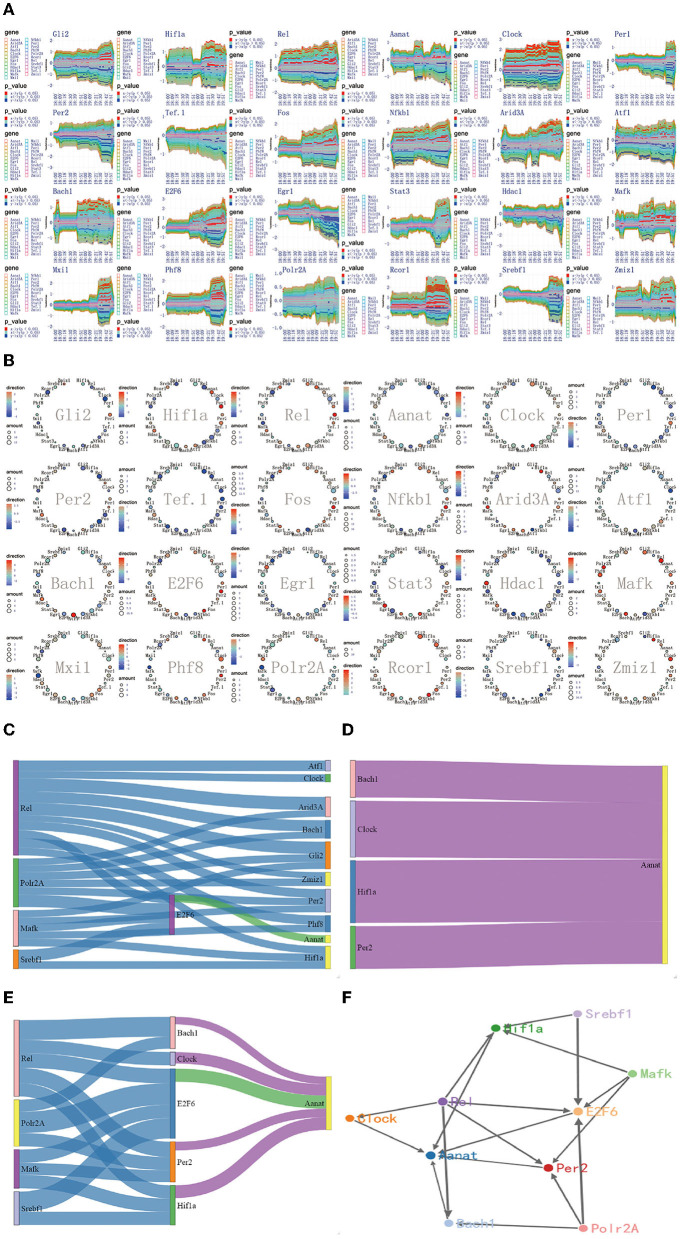
The regulatory mechanism of second extremely significant information transfer process in 18:00–20:00. **(A)** The time-dependent variation of transfer information among 24 circadian genes in 18:00–20:00. **(B)** The complete regulatory relationship among 24 circadian genes in 18:00–20:00. **(C)** The target genes regulated by transfer information from 14:00 to 16:00. **(D)** The rate-limiting gene *Aanat* is activated by *Hif1a, Bach1, Clock*, and *Per2* in 18:00–20:00. **(E)** The main mechanism for information transfer process in 18:00–20:00, *Hif1a, Bach1, Clock, E2f6*, and *Per2* are regulated simultaneously by 4 promoter genes, *Rel, Polr2A, Mafk*, and *Srbf1* and contribute genetic information to *Aanat*. **(F)** The targeting regulatory relations between promoter genes and *Aanat* in 18:00–20:00.

**Figure 8:**
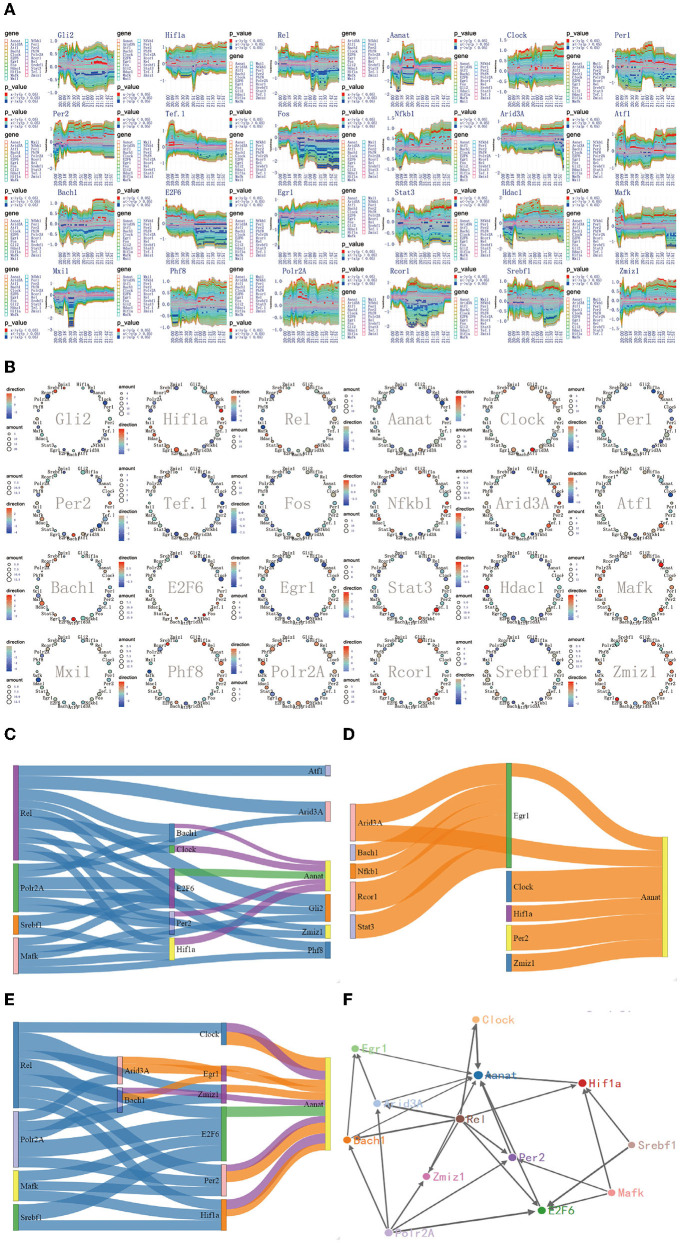
The regulatory mechanism of *Aanat* significant expression process in 20:00–22:00. **(A)** The time-dependent variation of transfer information among 24 circadian genes in 20:00–22:00. **(B)** The complete regulatory relationship among 24 circadian genes in 20:00–22:00. **(C)** The target genes regulated by transfer information from 18:00 to 20:00. **(D)** The rate-limiting gene *Aanat* is activated by *Hif1a, Bach1, Clock, Per2, Egr1*, and *Zmiz1* in 20:00–22:00. **(E)** The main mechanism for information transfer process in 20:00–22:00, *Hif1a, Bach1, Clock, E2f6, Per2, Egr1*, and *Zmiz1* are regulated simultaneously by 4 promoter genes, *Rel, Polr2A, Mafk*, and *Srbf1* and contribute genetic information to Aanat. **(F)** The targeting regulatory relations between promoter genes and *Aanat* in 20:00–22:00.

The first encoding method uses the diagram to represent the one-to-many relationship. This scheme taken on a circular layout, where the gene located in the center is the selected central gene and the remaining genes are distributed in a circle around. The transfer information between two genes is encoded as a circle located on the surrounding variables. The radius of the circle encodes the transfer entropy between two genes and the color encodes the direction of the information transferred between two genes. For example, given a transfer network with 3 node variables *A*, *B*, and *C*, we choose node *B* as central variable, and it is assumed that *TD*(*B, A*) is positive and *TD*(*B, C*) is negative, so the circle *A* in red and the circle *C* in blue. It is generally true that the darker the color, the more significant information transfer and the stronger the regulatory effect. Based on this coding model, we obtained the one-to-many regulatory relationship for each circadian gene to the other 23 circadian genes in the time periods of 14:00–16:00, 18:00–20:00, and 20:00–22:00, as shown in Figures 6B, 7B, 8B.

The second encoding method uses the sankey diagram to represent the information transfer pathways. According to the first encoding method, we have obtained the one-to-many relationship. In order to make the information transfer pathways easy to understand, the sankey diagram, integrating 24 one-to-many relationship is used. This scheme takes on a two-column rectangular layout. The genes on the left side act as promoter genes. The genes on the right side act as a receptor gene. Obviously, if promoter genes and receptor genes are specified, we can get all the information transfer pathways from promoter genes to receptor genes. Based on this coding model, we obtained all the information transfer pathways from the promoter genes *Rel, Polr2A, Mafk*, and *Srbf1* to the rate-limiting enzyme gene *Aanat* in the time periods 14:00–16:00, 18:00–20:00, and 20:00–22:00, as shown in Figures 6C–E, 7C–E, 8C–E.

The third encoding method uses a node-linked gene regulatory network. According to the diagram and the sankey diagram, we have obtained the information transfer pathways from the promoter genes *Rel, Polr2A, Mafk*, and *Srbf1* to the rate-limiting enzyme gene *Aanat*. In order to make these pathways more intuitive, we used a node-linked gene regulatory network. This scheme takes on a force-guided layout, which allows no masking between node genes after dynamic changes in the layout. In the regulatory network, we can clearly identify the key genes as well as the regulatory relationships between key genes and other genes. The network approach has been widely used in the study on the regulation mechanism of gene. Based on this coding model, we obtained different regulatory pathways for the rate-limiting enzyme gene *Aanat* in the 14:00–16:00, 18:00–20:00, and 20:00–22:00 time periods, as shown in Figures 6F, 7F, 8F.

### 3.4. Two Extremely Significant Information Transfer Processes Involved in Melatonin Synthesis

Using the method detailed in complete information transfer network among rhythmic genes, the time-dependent variation of transfer information between 24 circadian genes in 24 h is derived. As shown in Figure 5, two extremely significant information transfer processes are involved in melatonin synthesis. The first extremely significant information transfer process occurs in 14:00–16:00 (see Figure 5A). On November 15th, the sunset time is around 16:30. From 14:00 to 16:00, the light intensity was significantly decreased. In the laboratory notebooks, we find that there is no obvious natural light around 16:00. We therefore have gotten a very important conclusion: as the light intensity decreased, melatonin synthesis mechanism has started, which is embodied in circadian genes, *Rel, Polr2A, Mafk*, and *Srbf1* become active. Activated genes contribute genetic information to the next genes through various biological mechanisms. And transfer information reach peak around 15:10. This important process is most essential for *Aanat* gene expression. The study time on the synthesis mechanism of melatonin will be brought forward to 15:10 from 20:00. The second extremely significant information transfer process occurs in 18:00–20:00. In the laboratory notebooks, we found the experiment continued with no light source in 18:00–20:00. Results of this research demonstrate that relationship between rhythmic genes regulation in this stage is unaffected by the light intensity. Especially, the information transfer process has a very obvious two-way mechanism, as shown in Figure 5B. It demonstrates that this information transfer process is regulated by the first information transfer process. It has a very obvious cascade response in this process. By observing the expression trend of *Aanat* in Figure 2, it was clearly found that the expression level was low before 20:00, while increased obviously after 20:00 and peaked at midnight. These results demonstrate that the rate-limiting gene *Aanat* should be extensively activated in 18:00–20:00.

### 3.5. The Regulatory Mechanism of First Extremely Significant Information Transfer Process in 14:00–16:00

Using the method detailed in complete information transfer network among circadian genes (Figure 4), the time-dependent variation of transfer information between 24 circadian genes in 14:00–16:00 is derived. As shown in Figure 6A, we have gotten a very important conclusion: as the light intensity decreased, melatonin synthesis mechanism has started, which is embodied in circadian genes, *Rel, Polr2A, Mafk*, and *Srbf1* become active. Activated genes contribute genetic information to the next genes through various biological mechanisms. Using the integrated graphic programming, the regulatory relationship between 24 circadian genes in this time period can be clearly and directly displayed. As shown in Figure 6B, it is obvious that Rel regulates downstream target genes *Per2, Gli2, Hif1a, Clock, E2f6, Bach1*, and *Zmiz1*. *Polr2A* regulates downstream target genes *Zmiz1, Gli2, Per2, Hif1a, E2f6*, and *Rcor1*. *Mafk* regulates downstream target genes *Hif1a, Gli2, Per2, Clock*, and *E2f6*. *Srbf1* regulates downstream target genes *Hif1a* and *E2f6*.

Using the gene regulatory networks and sankey diagram, the targeting regulatory relations between 4 promoter genes and next genes can be clearly and directly obtained. Figure 6C represents the target genes regulated by 4 promoter genes. Figure 6D represents the rate-limiting gene *Aanat* only regulated by *E2f6* in this time period. By comparing those two pictures, we can see that *E2f6* is regulated simultaneously by four 4 promoter genes, and contribute genetic information to *Aanat* (see Figure 6E). In dynamic time-series expression profiles, we found that the average expression level of *Aanat* is 10.92 in 14:00–16:00. The experimental result is found to be in line with the above theoretical results. Due to the transfer information extraction is based on Hidden Markov Model from 480 dynamic time-series expression profiles (*P* < 0.05), the genes shared similar expression pattern are not necessarily acceptable targeting gene in information transfer process. For example, circadian genes *Per1* and *Per2* shared similar expression pattern (see Figure 2), but only *Per2* is was regarded as acceptable targeting gene. This result is consistent with previous study (Baba et al., 2015).

Furthermore, in addition to the main mechanism for information transfer process (see Figure 6E), the compensatory mechanism can be identified by in this work. For example, *E2f6* is regulated not only by *Rel, Polr2A, Mafk*, and *Srbf1*, but also by *Fos* and *Sata3* (see Figure 6B). Such results strongly suggest that the relationship between circadian genes is rather complex. The two mechanisms are recognized by using reasonable algorithm, scientific data and network model.

In 14:00–16:00, according to the multiplexed imaging analysis method, the targeting regulatory relations for the rate-limiting gene *Aanat* is described in Figure 6F. This extremely significant information transfer process provides the fundamental guarantee for the synthesis of melatonin.

### 3.6. The Regulatory Mechanism of Second Extremely Significant Information Transfer Process in 18:00–20:00

Using the method detailed in complete information transfer network among circadian genes (Figure 4), the time-dependent variation of transfer information between 24 circadian genes in 18:00–20:00 is derived. As shown in Figure 7A, it is immediately obvious discovery: The circadian genes, *Hif1a, Bach1, Clock*, and *Per2* are clearly regulated by the first significant information transfer process and obviously contribute genetic information to the next genes. This information transfer process has a very obvious two-way mechanism. Using the integrated graphic programming, the regulatory relationship between 24 circadian genes in this time period can be clearly and directly displayed. As shown in Figure 7B, it is obvious that *Hif1a* is regulated by *Rel* and *Polr2A* and contribute genetic information to *Aanat*. *Clock* is regulated by *Rel* and contribute genetic information to Aanat, *Bach1* is regulated by *Rel* and *Mark* and contribute genetic information to *Aanat, Per2* is regulated by *Gli2, Rel*, and *E2f6* and contribute genetic information to *Aanat*.

Using the gene regulatory networks and sankey diagram, the targeting regulatory relations between promoter genes and the rate-limiting gene *Aanat* can be clearly and directly obtained. Figure 7C represents of the transduction pathway from promoter genes to *Hif1a, Bach1, Clock*, and *Per2*. Figure 7D represents of the rate-limiting gene *Aanat* is regulated by *Hif1a, Bach1, Clock*, and *Per2* in this time period. By integrating those two processes, we can see that *Hif1a, Bach1, Clock, E2f6*, and *Per2* are regulated simultaneously by four 4 promoter genes, *Rel, Polr2A, Mafk*, and *Srbf1* and contribute genetic information to *Aanat* (see Figure 7E).

According to the multiplexed imaging analysis method, the targeting regulatory relations for the rate-limiting gene *Aanat* in this time period is described in Figure 7F. Compared with the regulatory relationship in 14:00–16:00, four pathways are added to activate the *Aanat* in 18:00–20:00. The activation pathways increased significantly, providing a mechanism guarantee that the *Aanat* gene expression in rat pineal gland can be markedly enhanced after 20:00.

### 3.7. The Regulatory Mechanism of Aanat Significant Expression Process in 20:00–22:00

Although four pathways are added to activate the *Aanat* in 18:00–20:00, *Aanat* mRNA expression is not enhanced before 20:00. The expression amount is increased significantly in 20:00–22:00 (see Figure 2). These results suggest two important contentions. The first is that it takes nearly 2 h from *Aanat* gene activated by signal transduction to transcription and translation products. This contention is supported in published study (Yao et al., 2005). The second is that there is additional activation pathway for rate-limiting gene *Aanat* activation. In order to support this contention, we delve into the targeting regulatory relations among circadian genes by using the method detailed in complete information transfer network among circadian genes (Figure 4). We obtain the time-dependent variation of transfer information between 24 circadian genes in 20:00–22:00, as shown in Figure 8A. From the figure, we can see that there is no obvious information transmission among circadian genes. Therefore, the regulation of *Aanat* gene expression is achieved through genetic regulatory systems, which are structured by transfer networks in 18:00–20:00.

By using the integrated graphic programming, we found that rate-limiting gene *Aanat* is activated not only by the four circadian genes (*Hif1a, Bach1, Clock*, and *Per2*) in 18:00–20:00 but also by two new circadian genes (*Egr1* and *Zmiz1*) in 20:00–22:00, as shown in Figure 8B. In order to clearly represent the information transfer process, we use the gene regulatory networks and sankey diagram to describe the magnitude and direction of regulation information. Figure 8C represents that *Hif1a, Bach1, Clock, E2f6*, and *Per2* are regulated simultaneously by four 4 promoter genes, *Rel, Polr2A, Mafk*, and *Srbf1* and contribute genetic information to *Aanat*. Figure 8D represents that the rate-limiting gene *Aanat* is regulated by *Hif1a, Bach1, Clock, Per2, Egr1*, and *Zmiz1* in 20:00–22:00. By integrating those two processes, we found that there are two new signal transduction pathways besides the four pathways in 18:00–20:00 (see Figure 8E).

According to the multiplexed imaging analysis method, the targeting regulatory relations for the rate-limiting gene *Aanat* in this time period is described in Figure 8F. Compared with the regulatory relationship in 18:00–20:00, there are six pathways to activate the *Aanat* in 20:00–22:00. This led to the significant expression of the rate-limiting gene *Aanat*, and peaked in midnight.

## 4. Conclusions

As we all know, melatonin regulates sleep and wake cycles, while disruption of its secretion can cause insomnia and fatigue. Scientists have identified that the synthesis and secretion of melatonin was highly correlated *Aanat* activity. And many circadian genes plays an important regulatory role in *Aanat* activity and melatonin synthesis. Thus, explore relationships among circadian genes which promise to reveal the regulation mechanism of melatonin synthesis while for the treatment of insomnia.

In this work, we propose a new omics sequencing method combined with a new data analysis method to discuss the problem of circadian rhythm. This method can well complete visualization of melatonin synthesis, especially the key targeting regulation relationship among circadian genes. It is a very important supplement to the widely used transcriptome sequencing technology and fills the whole variety trend of process ignored in the existing differential expression theory.

The new omics sequencing method obtained the dynamic time-series expression profile with 480 pineal gland samples in complete 24 h melatonin synthesis cycle. The highly multiplexed transfer information theory, combining novel visualization strategies, provides the asymmetric information in the signal transduction pathway of melatonin synthesis process. In addition, we also design a dynamic analytical model to present complete picture for melatonin synthesis and analysis of the targeted regulation among circadian genes. Our method visualized the whole process of melatonin synthesis associated complete targeting regulatory relations among circadian genes. Firstly, from 14:00 to 16:00, with the reduction of light intensity, *Rel, Polr2A, Mafk*, and *Srbf1* are activated and started the synthesis mechanism of melatonin. Since rate-limiting gene *Aanat* is only modulated by *E2f6* activated in this period, it doesn't resulted in high expression amount of *Aanat* gene. Therefore, this important startup process is rarely found in the current study. This important discovery makes study on the synthesis mechanism of melatonin in advance about 5 h. Secondly, from 18:00–20:00, the regulatory relationship between circadian genes has obvious two-way mechanism, The reason for this phenomenon is that the circadian genes *Hif1a, Bach1, Clock*, and *Per2* which are controlled by the promoter genes *Rel, Polr2A, Mafk*, and *Srbf1* further activates rate-limiting gene *Aanat*. Compared with the regulatory mechanism in 14:00–16:00, there are four additional pathways to activate the rate-limiting gene *Aanat*. The significant increase of activation pathway provides a mechanism guarantee for the significant expression of *Aanat* after 20:00. Thirdly, from 20:00 to 22:00, the rate-limiting gene *Aanat* is not only continuously regulated by the four circadian genes *Hif1a, Bach1, Clock*, and *Per2* but also increased additional regulatory factor of *Egr1* and *Zmiz1*, resulting in a significant increase in the expression amount.

Scientific experimental design and solid theoretical foundation are all crucial to effectively solve the biological questions. In view of the problem of melatonin synthesis and secretion in the rat's pineal gland, this process is characterized by dynamic changes in the 24-h rhythm cycle. Advanced multidisciplinary solutions, the integration of dynamic time-series transcriptomic technique and highly multiplexed transfer information theory with four visualization methods, are the blue sky of melatonin synthesis process. This integrated knowledge will help resolve many unanswered mechanisms about melatonin synthesis and insomnia problem. Currently, most landmark studies in the field demonstrate theoretical potential, with a growing number of studies resulting in critical biological insights into melatonin dyssynthesis. Broader uptake of dynamic time-series transcriptomic technique and analytical advancements using transfer information theory will undoubtedly lead to new insight of dyssomnia treatments.

## Data Availability Statement

The 480 dynamic sequential omics profiles supporting reported results are available for download at Sequence Read Archive (SRA) (SRR18934928 ~ SRR18935407) https://www.ncbi.nlm.nih.gov/sra/?term=SRR18934928; https://www.ncbi.nlm.nih.gov/sra/?term=SRR18934929; https://www.ncbi.nlm.nih.gov/sra/?term=SRR18934930 - https://www.ncbi.nlm.nih.gov/sra/?term=SRR18935406; https://www.ncbi.nlm.nih.gov/sra/?term=SRR18935407. The code, appendix data (Appendixs 1–3 in Supplementary Material) support the findings and other accessories and materials can be obtained through GitHub with no access limits (https://github.com/litao0401/circadian-gene-network).

## Ethics Statement

The animal study was reviewed and approved by Experimental Animal Welfare and Ethics Committee of Inner Mongolia Agricultural University.

## Author Contributions

TL and JY were involved in the concept and design. TL, SheW, YX, ShiW, HJ, CL, and YL contributed to the experiments. TL, ZL, and DZ contributed to the technical investigation. TL and YW analyzed the data. TL and ZL contributed to the writing original draft. TL and JC contributed to the writing—review and editing and acquired the funding. YZ and HZ contributed to the supervision. All authors contributed to the article and approved the submitted version.

## Funding

This work was funded by the Natural Science Foundation of Inner Mongolia Autonomous Region (2019MS03014 and 2021MS03091) and the Major Science and Technology Project of Inner Mongolia Autonomous Region (2019ZD016 and 2020ZD0007).

## Conflict of Interest

The authors declare that the research was conducted in the absence of any commercial or financial relationships that could be construed as a potential conflict of interest.

## Publisher's Note

All claims expressed in this article are solely those of the authors and do not necessarily represent those of their affiliated organizations, or those of the publisher, the editors and the reviewers. Any product that may be evaluated in this article, or claim that may be made by its manufacturer, is not guaranteed or endorsed by the publisher.
